# Exercise management in pediatric type 1 diabetes: a multicenter study of healthcare professionals’ perspectives

**DOI:** 10.1007/s00431-026-07472-7

**Published:** 2026-10-13

**Authors:** Hilla Reznik, Avivit Brener, Mohamad Sharkia, Marianna Rachmiel, Yonatan Yeshayahu, Ilana Zalmon-Koren, Lior Carmon, Dikla Pivko-Levy, Marie Mouler, Liat de Vries, Orit Pinhas-Hamiel, Floris Levy-Khademi, Michal Cohen

**Affiliations:** 1 The Ruth & Bruce Faculty of Medicine, Technion Israel Institute of Technology, Haifa, Israel; 2 The Institute of Pediatric Endocrinology, Diabetes, and Metabolism, Dana Dwek Children’s Hospital, Ichilov Hospital, Tel Aviv, Israel; 3 Pediatric Endocrinology and Diabetes Unit, HaEmek Medical Center, Afula, Israel; 4 Pediatric Endocrinology and Diabetes Institute, Shamir (Assaf Harofeh) Medical Center, Beer Yakov, Israel; 5 Department of Pediatrics, Samson Assuta Ashdod University Hospital, Ashdod, Israel; 6 Pediatric Endocrinology Unit, Lady Davis Carmel Medical Center, Clalit Health Services, Haifa, Israel; 7 Pediatric Endocrinology Unit, Soroka Medical Center, Beer-Sheva, Israel; 8 Pediatric Endocrine and Diabetes Unit, E. Wolfson Medical Center, Holon, Israel; 9 The Jesse Z and Sara Lea Shafer Institute for Endocrinology and Diabetes, National Center for Childhood Diabetes, Schneider Children’s Medical Center of Israel, 4920235 Petach Tikva, Israel; 10 Pediatric Endocrine and Diabetes Division, Edmond and Lily Safra Children’s Hospital, Chaim Sheba Medical Center, Ramat Gan, Israel; 11 Division of Pediatric Endocrinology, Department of Pediatrics, Shaare Zedek Medical Center, Jerusalem, Israel; 12 Pediatric Endocrinology and Diabetes Unit, The Ruth Rappaport Children’s Hospital, Rambam Health Care Campus, Haifa, Israel; 13 School of Medicine, Gray Faculty of Medical & Health Sciences, Tel Aviv University, Tel Aviv, Israel; 14 Faculty of Health Sciences, Ben-Gurion University of the Negev, Beer Sheva, Israel; 15 The Faculty of Medicine, Hebrew University, Jerusalem, Israel

**Keywords:** Exercise, Education, Pediatrics, Type 1 diabetes, Barriers

## Abstract

Regular exercise is an important component of care for children with type 1 diabetes (T1D), supporting both physical and psychological health. However, many do not meet recommended activity levels, and exercise does not consistently improve glycemic control. Exercise management in T1D is complex and influenced by multiple factors, including child, activity, and treatment characteristics. These challenges highlight the importance of comprehensive exercise-directed guidance (EDG). This study aimed to characterize EDG practices in pediatric T1D and identify key challenges faced by healthcare professionals (HCPs). Physicians, nurses, and dietitians from 11 pediatric diabetes centers participated. Participants completed anonymized electronic questionnaires assessing current practices, content, and perceived challenges related to EDG. Response rate was 87.2%; 130 HCPs participated; all reported providing EDG to patients. Considerable variability was observed in the timing, content, and educational resources used. While the majority (85.9%) reported offering guidance to all patients, others indicated guiding only when specific needs arose. The most frequently addressed topics included the immediate effects on blood glucose, adjusting carbohydrate intake, and general preparations for exercise. Less frequently discussed topics were planning exercise regimens and encouraging involvement of family or peers. Challenges by order of prevalence were insufficient teaching aids (42.3%), EDG complexity (40.0%), visit duration (36.2%), and knowledge gaps (33.1%).

*Conclusion*: Although HCPs acknowledge the importance of exercise in pediatric T1D and incorporate EDG into clinical care, substantial variability exists in its delivery. Improving the availability and usability of educational tools and strengthening social support components may enhance exercise education for children with T1D.

**Table:** 

**What is Known:** • *Exercise-directed guidance (EDG) is essential for children with type-1-diabetes, yet challenging to implement.* • *Knowledge of barriers to EDG is growing, but evidence remains limited across healthcare settings and professional groups.*
**What is New:** • *In this national multicenter study of healthcare professionals, all participants reported providing EDG, yet its timing,content, and availability of educational tools varied.* • *Limited teaching aids, the complexity of EDG, and time constraints were the main barriers; addressing family and peer involvement and exercise-specific planning were identified as areas for improvement.*

**Supplementary Information:**

The online version contains supplementary material available at https://doi.org/10.1007/s00431-026-07472-7.

## Introduction

Exercise refers to planned, structured, and repetitive bodily movement performed to improve or maintain components of physical fitness or skills [1]. It is also performed to promote enjoyment and foster connections with others through games and group activities. Exercise offers numerous health benefits, including improved cardiovascular health, increased insulin sensitivity, strengthened muscles and bones, and reduced chronic disease risk [2, 3]. Improvement in quality of life, well-being, sense of belonging, and self-esteem is also associated with the regular performance of exercise [2, 4, 5]. Many of these benefits address specific areas of health risk in individuals with diabetes. Type 1 diabetes (T1D), the most common form of diabetes in children and adolescents, is characterized by autoimmune destruction of insulin-producing pancreatic beta cells and results in lifelong dependence on exogenous insulin. T1D requires careful management of blood glucose levels, insulin dosing, and carbohydrate intake [6].

Exercise has the potential to improve glycemic control in individuals with diabetes [7–10]. However, this might be impaired by the complexity of effects of exercise on glucose in T1D [11, 12]. These are influenced by multiple interacting factors, including the type of exercise (aerobic, anaerobic, or mixed), its intensity and duration, the insulin dose and timing, and carbohydrate intake and its timing [12, 13]. Individual characteristics also influence the glycemic response to exercise, including age, insulin sensitivity, physical fitness, muscle and fat mass, and stress level (e.g., competition versus routine practice). At least 60 min of moderate-intensity or vigorous daily activity, with resistance activity incorporated at least 3 times per week, is recommended in children with T1D [12, 13], similar to the recommendations in the general pediatric population [14]. Despite recommendations and the multiple benefits of activity, studies suggest that children with T1D perform less than the recommended daily activity and that they are less active than their peers without diabetes [15]. Fear of both hypoglycemia and hyperglycemia is a common concern and a major barrier to the performance of exercise in T1D [16–19]. Suboptimal knowledge and difficulties in the implementation of recommendations have also been reported as challenges [20, 21]. To address these concerns and allow the safe performance of exercise, specific counseling provided by the diabetes clinic team is key. Such counseling should be individualized to address the needs of each child and family.

Exercise counseling can be challenging, and its delivery is often complex and time-consuming [22]. In recent years, a growing number of studies have addressed the specific needs, concerns, and perspectives of healthcare professionals (HCPs) regarding exercise-directed guidance (EDG) in T1D [21, 23–25]. While HCPs report awareness of guidelines in the field and recognize their responsibility to support exercise in pediatric T1D, they report recurring barriers to doing so effectively. Interviews with 11 HCPs caring for children with diabetes from across the UK describe limited consultation time and difficulty translating general exercise recommendations into practical, family-friendly advice [25]. Together, a systematic review of the literature on this topic, based mostly on qualitative studies, predominantly from the UK, with additional studies from Ireland, Australia, Brazil and Sweden, and two more recent surveys of HCPs caring for children with diabetes—a UK national survey and an International Society for Pediatric and Adolescent Diabetes (ISPAD)–based international survey—further highlight important barriers. Insufficient knowledge, time, and resources as well as the complexity of guidance appears to be the major obstacles reported by HCPs [21, 23, 24]. Moreover, in a recent qualitative study from the UK, involving a relatively small number of adolescents, parents, and HCPs, families reported that clinical guidance was often generic, limited, or absent leaving families to rely on their own trial-and-error strategies [20]. These studies have greatly advanced the understanding of the challenges faced by HCPs in providing exercise counseling to children with T1D. While qualitative studies allow in-depth exploration of the topic, their small sample size may limit generalizability. Surveys, in contrast, may be subject to recruitment bias, as HCPs who feel more confident in their counseling may be more likely to participate. Comparable challenges have been reported among HCPs caring for adults with T1D in studies from the UK and Australia [26, 27]. Differences between countries in clinic staffing models, the roles of HCPs across different professions, referral pathways, and cultural attitudes toward chronic illness may shape both the barriers to effective EDG in T1D and the strategies available to address them. Data from additional countries and healthcare settings would be valuable in improving the generalizability of information and could assist in highlighting areas for future enhancement.

Building on the available body of evidence, we designed a nationwide study incorporating HCPs from multiple pediatric diabetes clinics and the major professional disciplines involved in EDG. In this study, we aimed to identify and characterize strengths and challenges of the EDG provided by HCPs in pediatric diabetes clinics, specifically regarding (A) timing and frequency, (B) content of guidance and the available educational tools, and (C) strengths and challenges reported by HCPs, and in addition, (D) we aimed to evaluate the internal consistency of the study questionnaire.

## Materials and methods

### Subjects

This was a cross-sectional multicenter questionnaire-based study. All 13 larger pediatric diabetes clinics in Israel, defined as academic, university-affiliated centers, approved for pediatric endocrinology fellowship training, were invited to participate. At each center, a local co-investigator introduced the study to eligible HCPs, in staff meetings. Participation was voluntary. Physicians, nurses, and dietitians treating children with T1D were eligible to participate in the study. These three professional groups were selected as they are universally present across all participating centers and are consistently involved in the provision of EDG in pediatric T1D clinics in Israel. Other professionals who may contribute to exercise counseling in some settings (e.g., exercise physiologists, psychologists, social workers) are not routinely part of pediatric diabetes teams in this setting and were therefore not included. Participants completed an anonymized digital questionnaire. Research was conducted in accordance with the Declaration of Helsinki. Ethics approval was granted before commencing the study. The study was approved by the coordinating (Rambam Healthcare Center) site’s institutional Helsinki committee, and subsequently, each additional participating site’s institutional Helsinki committee granted local approval. All approvals were obtained prior to study initiation and participant recruitment. Informed consent to participate was obtained from all participants prior to study enrollment. The institutional Helsinki committee at two participating medical centers waived the requirement for written informed consent and approved the use of verbal informed consent. At the other nine participating centers, written informed consent was obtained from all participants.

### The study questionnaire (Supplementary)

The study questionnaire was developed by HR and MC based on guidelines, expert opinions, and consensus statements regarding exercise in pediatric T1D [12, 13, 22]. The co-investigators reviewed the questionnaire for face validity; edits based on their comments were introduced prior to commencing the study. The questionnaire assessed the following areas: (1) participant and clinic characteristics: gender, role and seniority, the clinic size, and ethnic distribution; (2) timing and frequency of the EDG; (3) characteristics of EDG, including the educational tools available and content of teaching—participants were asked to rate on a scale of 1 to 3 (1 “usually,: 2 “sometimes,” 3 “usually not”) how often they address each of 13 specific teaching topics; and (4) strengths and challenges of EDG as experienced by the HCPs. Participants were asked to rate on a scale of 1–4 (1 “significant,” 2 “moderate,” 3 “minimal,” 4 “not at all”), four potential barriers: availability of instructional materials, the complexity of the training, the time allowed for clinic visits, and their personal knowledge gaps. They then had the option to express challenges and strengths using free text and add comments. Prior to study initiation, the questionnaire was pilot-tested by two of the co-investigators to identify any technical issues and to evaluate the time required for completion. Formal cognitive interviews were not conducted, but open-ended free-text questions allowed participants to highlight any items and topics they found unclear. The internal consistencies of the 13-item EDG content scale and the 4-item barriers to EDG scale were subsequently evaluated using Cronbach’s alpha coefficient and item–total correlations.

### Statistical analysis

Statistical analysis was performed using the Statistical Package for Social Sciences Software (IBM SPSS Statistics, version 28.0). Categorical variables are expressed as frequencies and proportions. Comparisons based on profession (physician, nurse, dietitian) were conducted using chi-square tests. A *p* value < 0.05 was considered significant. The internal consistency of the multi-item questionnaire scales was evaluated using Cronbach’s alpha coefficient; an alpha > 0.7 was considered acceptable.

## Results

### Study participants (Table 1)

**Table 1:** Participant and clinic characteristics

		All (*n* = 130)	Physicians (*n* = 71)	Nurses (*n* = 31)	Dietitians (*n* = 28)	*p* value
Female gender^*^		110 (84.6%)	51 (71.8%)	31 (100%)	28 (100%)	< 0.001
Experience in pediatric diabetes (years)	< 5	36 (28.1%)	19 (27.1%)	8 (25.8%)	9 (33.3%)	0.564
	5–10	35 (27.3%)	20 (28.6%)	6 (19.4%)	9 (33.3%)	
	> 10	57 (44.5%)	31 (44.3%)	17 (54.8%)	9 (33.3%)	
Clinic population (number of patients)	< 200	8 (6.2%)	3 (4.2%)	3 (9.7%)	2 (7.1%)	0.335
	200–600	90 (69.2%)	47 (66.2%)	24 (77.4%)	19 (67.9%)	
	600–1000	10 (7.7%)	6 (8.5%)	3 (9.7%)	1 (3.6%)	
	> 1000	22 (16.9%)	15 (21.1%)	1 (3.2%)	6 (21.4%)	
Time allowed for clinic visit (minutes)^*^	< 20	17 (13.2%)	3 (4.2%)	12 (38.7%)	2 (7.1%)	< 0.001
	20–40	99 (76.2%)	63 (88.7%)	18 (58.1%)	18 (64.3%)	
	> 40	14 (10.9%)	5 (7.0%)	1 (3.2%)	8 (28.6%)	

Data for all participants and per healthcare profession (physician, nurse, dietitian). Comparisons based on profession conducted using chi-square tests are presented. Results are presented as numbers and percentages of responders

^*^p < 0.05

Eleven out of 13 eligible clinics agreed to take part in the study, with 149 potential responders overall. Response rate was 87.2%, with 130 HCPs participating in the study, 71 physicians, 31 nurses, and 28 dietitians. One hundred and eight (84.6%) were females. Fifty-seven (44.5%) reported more than 10 years of experience in pediatric diabetes, and most (90, 69.2%) indicated working in clinics serving between 200 and 600 patients with T1D. The typical duration allowed for clinic visits was reported to range between 20 and 40 min for the majority of participants (99, 76.2%). Differences based on profession (physician, nurse, dietitian) are detailed in Table 1.

### Characteristics of EDG

#### EDG routines and tools (Table 2)

**Table 2:** Timing and frequency of the EDG and availability of teaching aids

Questions presented to participants Do you:	All (*n* = 130)	Physicians (*n* = 71)	Nurses (*n* = 31)	Dietitians (*n* = 28)	*p* value
Provide EDG in clinic?	130 (100%)	71 (100%)	31 (100%)	28 (100%)	NA
Provide EDG to all patients?	110 (85.9%)	58 (82.9%)	29 (93.5%)	23 (85.2%)	0.546
Provide EDG to competitive athletes?^*^	80 (62.5%)	43 (61.4%)	25 (80.6%)	12 (44.4%)	0.017
Provide EDG during the initial visits	85 (65.9%)	43 (61.4%)	22 (71.0%)	20 (71.4%)	0.507
Routine periodic EDG	73 (56.6%)	44 (62.9%)	18 (58.1%)	11 (39.3%)	0.102
Upon request	26 (20.2%)	15 (21.4%)	5 (16.1%)	6 (21.4%)	0.814
Have teaching aids available?^*^	84 (65.1%)	41 (57.7%)	26 (83.9%)	17 (63.0%)	0.038

Data for all participants and per healthcare profession (physician, nurse, dietitian). Comparisons based on profession conducted using chi-square tests are presented. Results represent the number (and percentage) of participants that provided a positive (yes) response to each of the detailed yes–no questions

^*^p < 0.05

All participants reported offering EDG to patients with T1D. One hundred and ten (85.9%) reported routinely guiding all patients, while 20 (15.4%) guide only specific children, including children who appeared to need a lifestyle change for health or social reasons, patients identified by the caregiver as mentally and emotionally ready to receive EDG, or patients who reported not to engage in any exercise. HCPs’ schedules for educational sessions varied; 85 (65.9%) participants indicated they provide the guidance during the first clinic visits following T1D diagnosis. The first clinic visit usually follows a hospital admission during which the family meets the team and is provided with comprehensive T1D education. Seventy-three participants (56.6%) keep a routine of periodic teaching. Eighty-four participants (65.1%) reported having educational materials available in clinic, with significant profession-based differences. Specifically, nurses more frequently reported having teaching aids available (83.9%) compared with dietitians (63.0%) and physicians (57.7%). The other characteristics did not differ when comparing professions.

#### EDG content (Fig. 1)

**Fig. 1:**
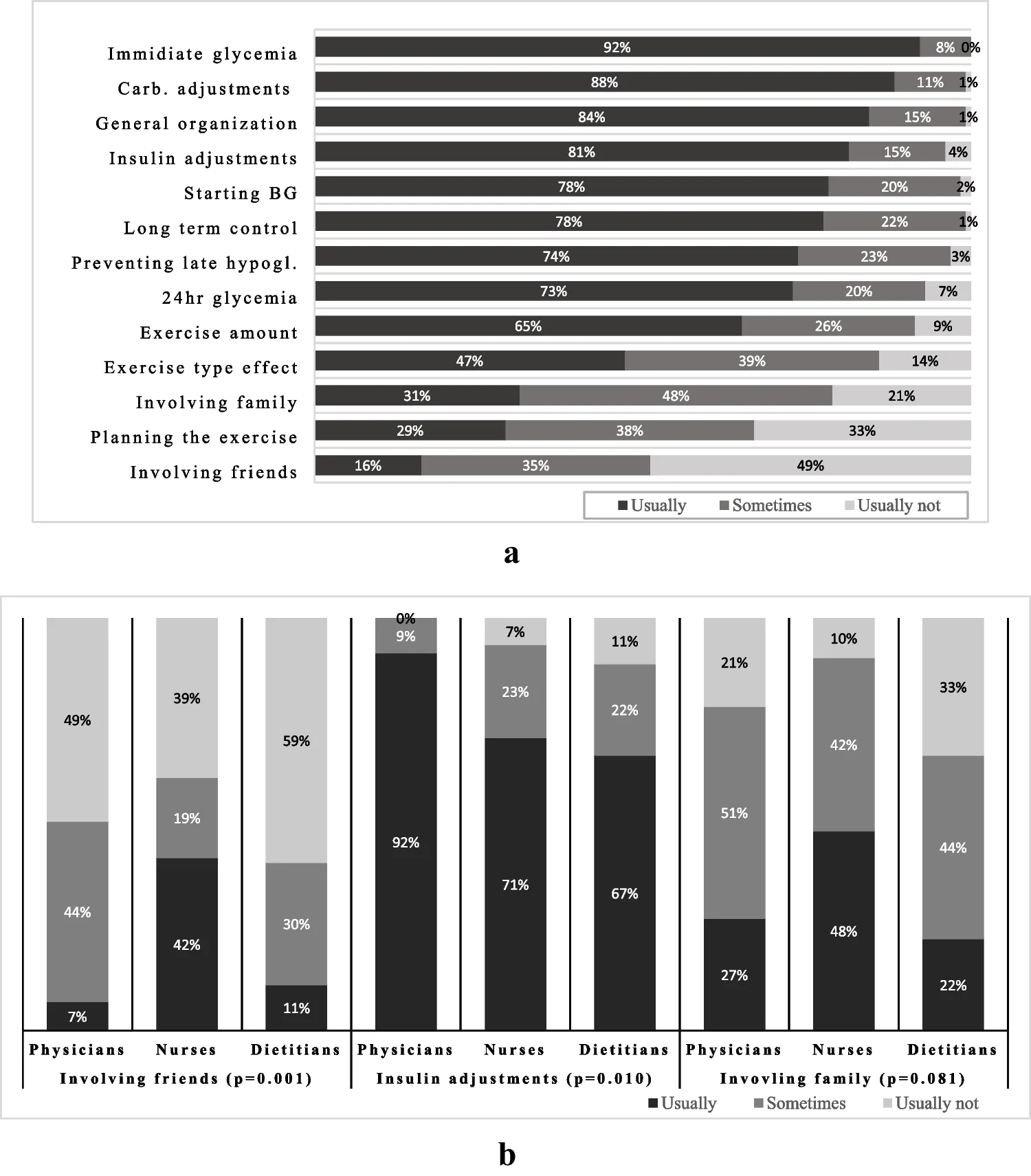
Reported prevalence of topics included in the EDG. a The frequency of addressing various EDG topics by all study participants. The frequency with which each of 13 specific topics is discussed during EDG as reported by HCPs. The percent of responders that chose each category is presented. Colors represent responses: black—usually yes, dark gray—sometimes, light gray—usually not. Carb—carbohydrate, BG—blood glucose. b EDG topics reported to be addressed at different frequencies between professions. The figure presents the three topics that differed significantly or trended to differ among professions. The percent of responders that chose each category is presented. Colors represent responses: black—usually yes, dark gray—sometimes, light gray—usually not

When asked about the frequency of including specific topics in EDG, the three most prevalent topics reported as “usually included” were the immediate glycemic effects (119, 92.2%), adjusting carbohydrates (114, 88.4%), and general diabetes-related preparations before exercise (109, 84.5%). These frequencies did not differ significantly between physicians, nurses, and dietitians. The three factors reported to be included in EDG the least often were involving friends in exercise or planning exercise (21, 16.3%), planning the type of exercise to address glucose control (37, 29.1%), and involving family members (40, 31.3%). We found significant differences between professions in the frequency with which they “usually” discuss involving friends in exercise planning and performance (41.9% of nurses compared with 11.1% of dietitians and 7% of physicians, *p* = 0.001) and in the frequency with which they addressed insulin adjustments in training sessions (71.0% of nurses compared with 66.7% of dietitians and 91.5% of physicians, *p* = 0.010). In addition, we found a trend toward a difference between professions in the frequency with which they involve family in exercise planning and execution (48.4% of nurses compared with 22.2% of dietitians and 27.1% of physicians, *p* = 0.081).

### Barriers to EDG (Fig. 2)

**Fig. 2:**
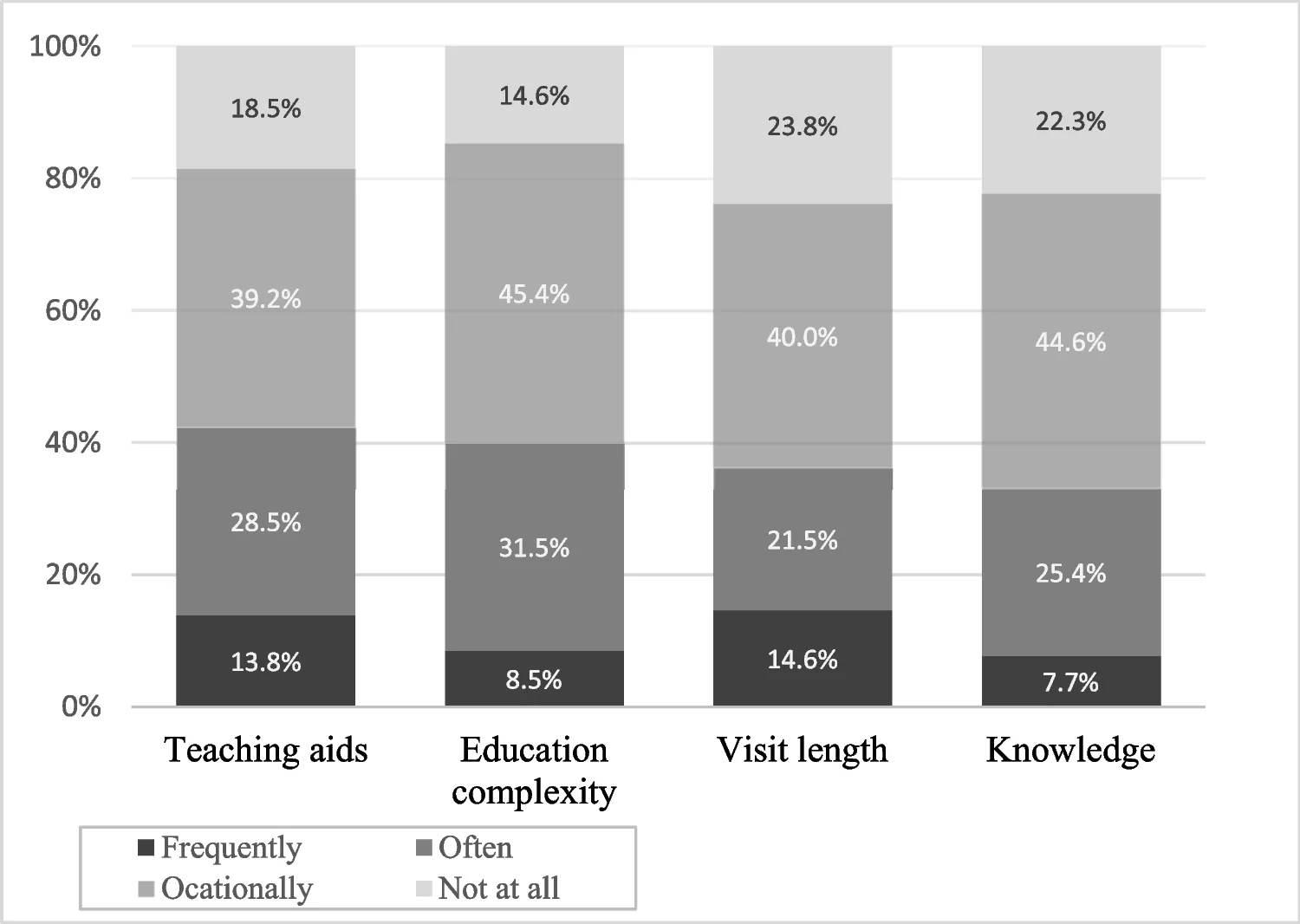
Perceived EDG barriers. The frequency with which each of the four EDG topics was reported as a barrier. The percentage of responders that chose each category is presented. Black—frequently, light gray—often, dark gray—occasionally, very light gray—not at all

Similar proportions of participants rated the four potential barriers to EDG presented in the questionnaire, as difficulties they encounter either frequently or often: availability of teaching aids (55, 42.3%), educational complexity (52, 40.0%), visit duration (47, 36.2%), and knowledge gaps (43, 33.1%). These did not differ significantly based on profession. Additional barriers reported in the free-text responses were difficulties in motivating patients, the need for information aids in multiple languages, and addressing the needs related to specific exercise types; the latter was the most prevalent free-text barrier mentioned by 7.4% of participants. Specific knowledge gaps that were mentioned by participants were related to assisting competitive athletes and adapting exercise to patient age.

### Validity of the study questionnaire

Evaluating the internal consistency of the 13-item “EDG content” scale yielded a Cronbach’s alpha coefficient of 0.782, suggesting good reliability for this questionnaire section. Item–total correlations ranged from 0.22 to 0.58, indicating that items made acceptable-to-good contributions to the overall scale. Evaluating the internal consistency of the 4-item “barriers to EDG” scale yielded a Cronbach’s alpha coefficient of 0.710, suggesting good reliability for this questionnaire section as well. Item–total correlations ranged from 0.30 to 0.52, indicating that items made acceptable-to-good contributions to the overall scale. No significant difficulties regarding the questionnaire were raised by participants.

## Discussion

In this multicenter national study, we aimed to characterize current pediatric T1D EDG practices as reported by HCPs. Three main findings emerged: HCPs across professions uniformly report offering EDG as part of routine care, addressing a range of essential topics in their counseling, particularly those related to immediate glycemic management; substantial variability exists in EDG routines and content; and key barriers to EDG provision were identified.

Our finding that all participants reported that they provide EDG to patients was reassuring and given the high participation rate likely reflects genuine HCP engagement in EDG. It may suggest that HCPs view the provision of EDG to be an integral part of their clinical responsibilities. This aligns with data from a survey conducted across pediatric clinics in England and Wales, which similarly found a high proportion of HCPs perceived EDG to be part of their clinical role [24]. The increasing availability of detailed international guidelines and published practical EDG approaches in recent years and their increasing discussion at professional meetings may explain this high proportion [12, 13, 28, 29]. However, it may not necessarily translate into familiarity with guideline content—in one survey, only 54% of HCPs reported being familiar with current recommendations [23]. Notably, the proportion of HCPs involved in EDG in our study was higher than that reported in a survey of HCPs caring for adult T1D patients in Australia, where less than half reported routinely discussing exercise in their consultations [27]. Whether this difference reflects an increased need for such guidance in younger patients, local or other factors, remains to be determined.

Despite the increased involvement in EDG, we identified variability in the timing and periodicity of guidance, with only 65.9% reporting inclusion of EDG in initial visits and only 56.6% reporting the provision of routine periodic EDG. This did not differ between professions. While some variability can be expected based on individual HCP approaches and on patient preferences and receptiveness, follow-up consultations are highly valuable, appreciated by patients and recommended in clinical guidelines [13, 30]. During clinical sessions, HCPs address varying patient needs and barriers, as well as different levels of understanding. Planned periodicity can allow for a stepwise approach, gradually increasing the depth of discussion and troubleshooting issues that might come up after a patient has implemented recommendations from a previous visit, thus better personalizing EDG [26, 31]. Such a staged approach may also address the repeatedly reported lack of sufficient time as a barrier to EDG [23, 24, 26].

We also found variability among participants in the content of EDG. In our cohort, “medical” topics such as the short-term glycemic response, carbohydrate and insulin adjustments, and safety precautions were reported to be more consistently addressed in EDG, than the more “exercise-specific” and social topics. This gap is consistent with prior work showing that family and peer support is perceived by children with T1D and their families as essential for exercise performance [18, 21, 25, 30]. Furthermore, research indicates that family members may experience anxiety when children with T1D engage in exercise, leading to reduced encouragement and fear of exercise among the children themselves [21]. Actively involving family members in the EDG process and addressing their concerns may help alleviate these fears and improve overall support, a strategy also acknowledged by HCPs [21]. In our study, exercise-specific themes were part of routine EDG for only a portion of participants, including the amount of exercise recommended (65%) and the effects of different types of exercise (47%). It should be noted that we assessed only whether participants addressed the topic of exercise amount, not the specific recommendations given—an area in which limitations in HCP knowledge have been reported [23]. Similar difficulty in discussing the effects of specific types of exercise has been reported by both children with T1D and HCPs in several studies [24, 26].

In our study, the availability of teaching aids for EDG emerged as a further area of variation and concern among participants. Nurses reported higher availability of teaching aids compared with dietitians and physicians, suggesting that lack of awareness of locally available tools by the latter two groups may be a contributing factor. A specific request for multilanguage information materials was highlighted by participants, reflecting the diversity of patient populations seen in clinics. The need for better educational tools is in keeping with findings in several studies in children and adolescents with T1D [23, 24]. EDG aids can also address the important barriers we identified, i.e., the complexity of teaching and limited time in clinic. These tools must be kept up to date with current knowledge and particularly with the changing technological landscape of T1D treatment. Enjoyable age-appropriate EDG materials, available for patients and families to revisit when questions arise, can further enhance understanding and independence while remaining time-efficient [25, 32]. A structured exercise education intervention for youth newly diagnosed with T1D and their families was accepted with great interest and positive feedback [33]. Education for the healthcare team should also be highlighted. Studies underscore the necessity of specialized training materials for HCPs conducting EDG for children with T1D [21, 24]. Such tools have been demonstrated to enhance HCP confidence and competence, enabling them to deliver more effective and individualized coaching in adults with T1D [34]. Based on our findings, we suggest that consistency in EDG may be enhanced by developing shared resources, potentially on a national scale, as well as through designing training programs for HCPs. Standardization of protocols and EDG materials can promote a more uniform approach within a clinical team. Responsibilities can be divided among HCPs so that different aspects of EDG are covered efficiently, or alternatively, a more redundant strategy can be adopted, in which key messages are reinforced by different HCPs. Either option could help address the lack of team support and inconsistent approaches previously reported by HCPs as barriers to EDG [24].

Our study has several limitations. The voluntary nature of participation may have introduced a selection bias; however, given the high response rate, we believe this is unlikely to significantly affect the results. Another limitation relates to the study design, which relied on self-reporting by HCPs, potentially biased by their perception of the “ideal” EDG. While direct observation of clinic visits could provide more accurate data, such an approach would be resource-intensive and may introduce a bias due to participants’ awareness of being observed. Another limitation of our questionnaire is that it did not address HCPs’ approaches to transitions between diabetes technologies, a topic that often requires specific modification of exercise counseling. Several factors may threaten the generalizability of the results: first, the study included only larger, university-affiliated centers, which may not represent smaller clinics where resources and practices may differ. Second, as this study was conducted in a single country, findings may not generalize to healthcare systems with different structures or resources. Finally, the predominance of female respondents reflects the gender composition of the pediatric diabetes workforce in Israel; however, it may limit generalizability to settings with a different gender distribution among healthcare providers.

## Implications for practice and future research

Our findings support the value of a structured, staged approach to EDG, beginning at diagnosis and revisited during follow-up. Shared, up-to-date, age-appropriate resources for families and concise guidance for HCPs, available in relevant languages, may help address the complexity of exercise counseling and limited consultation time. The lower frequency of addressing family and peer involvement also highlights an opportunity to incorporate broader social and behavioral support. Future research could evaluate whether standardized EDG tools and HCP training improve the consistency and quality of counseling and support safe participation in physical activity among children and adolescents with T1D. This aligns with previous calls for more accessible EDG resources and with emerging international initiatives to develop standardized counseling tools for children with T1D, their families, and HCPs.

## Conclusions

Our study had a high participation rate, enabling a broad overview of current EDG practices across pediatric T1D centers nationally. Although HCP involvement in EDG was universal in our cohort, we identified substantial variability in its timing, content, and available educational tools. The complexity of EDG and limited availability of educational aids were important barriers reported by approximately 40% of participants. Overall, our findings highlight opportunities to improve the consistency and accessibility of EDG and support more standardized approaches to EDG for children and adolescents with T1D.

## Supplementary Information

Below is the link to the electronic supplementary material.

ESM 1 (DOCX 32.4 KB)

## Data Availability

No datasets were generated or analysed during the current study.
