# Supplementary material for: Exercise management in pediatric type 1 diabetes: a multicenter study of healthcare professionals’ perspectives
Source: Eur J Pediatr. 2026 Oct 13;185(11):842. doi: 10.1007/s00431-026-07472-7 (PMC13661701; doi:10.1007/s00431-026-07472-7)
Supplement: Supplementary file 1 [file 431_2026_7472_MOESM1_ESM.docx]

**Healthcare Professional Questionnaire: Exercise-Directed Guidance for Children with Type 1 Diabetes**

1. **Role:**

- Senior physician
- Fellow
- Nurse
- Dietitian

2. **Gender:**

- Male
- Female

3. **Years of experience working in a diabetes clinic:**

- Less than 5 years
- 5–10 years
- More than 10 years

4. **What is your diabetes care setting?**

- Hospital
- Outpatient Clinic
- Private Practice
- Other

If you selected 'Other', please specify: ____________________________

5. **Approximately how many pediatric patients with type 1 diabetes are followed at your clinic?**

- Up to 100
- 100–200
- 200–600
- 600–1000
- More than 1000

6. **The ethnic background of the population in your clinic is:**

- Mixed
- Mainly Jewish
- Mainly Arab
- Other

If you selected 'Other', please specify: ____________________________

7. **Average time allocated for a patient visit in your clinic:**

- Up to 20 minutes
- 20–40 minutes
- More than 40 minutes

8. **Do you provide exercise counseling to individuals with type 1 diabetes?**

- Yes
- No

9. **Whom do you provide guidance to?**

- All patients
- Only those participating in regular exercise \ sports
- Other

If you selected 'Other', please specify: ____________________________

10. **Do you provide guidance to children with type 1 diabetes who engage in competitive sports?**

- Yes
- No

11. **When do you typically provide guidance on physical activity to children with type 1 diabetes? (Select all that apply)**

- During the initial clinic visits after diagnosis (within the 1^st^ month)
- Periodically for every patient
- Upon patient request
- Other

If you selected 'Other', please specify: __________________

If you selected 'Periodically for every patient', please specify: __________________

12. **To what extent do you address the following topics during guidance? (For each topic choose 'usually', 'Sometimes' or 'usually not')**
1) The amount of activity recommended for the patient: usually yes / Sometimes / Usually not
2) Personalized recommended blood glucose level before starting exercise: usually yes / Sometimes / Usually not
3) The immediate effect of exercise on blood glucose (during and immediately after activity): usually yes / Sometimes / Usually not
4) The effect of exercise on glucose levels during the 24 hours after activity: usually yes / Sometimes / Usually not
5) The long-term benefits of exercise performance: usually yes / Sometimes / Usually not
6) The glycemic effects of different exercise types (aerobic, anaerobic, mixed)
7) Preparation for activity - equipment and nutrition (e.g. equipment and glucose for treating hypoglycemia, insulin, a glucometer): usually yes / Sometimes / Usually not
8) Preparation for activity - insulin dose adjustments: usually yes / Sometimes / Usually not
9) Preparation for activity – using carbohydrates or other food: Usually yes / Sometimes / Usually not
10) Planning a workout / training session in terms of exercise type and order (aerobic, anaerobic, mixed): Usually yes / Sometimes / Usually not
11) Prevention of delayed hypoglycemia: Usually yes / Sometimes / Usually not
12) Involvement of family members in activity planning or performance: Usually yes / Sometimes / Usually not
13) Involvement of friends in activity planning or performance: Usually yes / Sometimes / Usually not
13. **Does your clinic have educational material or supporting resources (e.g., a handout or flyer) on the topic of exercise in type 1 diabetes that can be given to patients?**

- Yes
- No

**If you replied 'yes', is it in** Print / Electronic / Other

Please elaborate: ____________________

14. **Do you refer patients to any other information source on exercise in type 1 diabetes?**

- Yes
- No

If yes, please elaborate: _________________

15. **To what extent do you think the guidance you provide includes discussion of the individuals' barriers to exercise?**

- To a great extent
- To a moderate extent
- To a small extent
- Not at all

16. **To what extent do you offer solutions to cope with those barriers?**

- To a great extent
- To a moderate extent
- To a small extent
- Not at all

17. **To what degree do you experience the following topics as barriers to providing exercise counseling? Rate each topic as: significant, moderate, minimal, not at all**
1) Limited duration of clinic visit
2) Lack of sufficient knowledge
3) The complexity of recommendations
4) Lack of available teaching aids for exercise directed guidance
18. **Do you experience any other barriers to providing exercise counseling to individuals with type 1 diabetes? (Free text question) ____________________________**
19. **If you indicated that you lack sufficient knowledge to provide exercise guidance for individuals with type 1 diabetes, please specify: (Free text question) ____________________________**
20. **Additional comments: ______________________**
